# High Throughput Label Free Measurement of Cancer Cell Adhesion Kinetics Under Hemodynamic Flow

**DOI:** 10.1038/srep19854

**Published:** 2016-01-27

**Authors:** Adrianne Spencer, Aaron B. Baker

**Affiliations:** 1 University of Texas at Austin, Department of Biomedical Engineering, University of Texas, Austin, TX USA; 2 Institute for Cellular and Molecular Biology, University of Texas, Austin, TX USA; 3 Institute for Computational Engineering and Sciences (ICES), University of Texas, Austin, TX USA

## Abstract

The kinetics of receptor-mediated cell adhesion to extracellular matrix and adherent cell monolayers plays a key role in many physiological and pathological processes including cancer metastasis. Within this process the presence of fluidic shear forces is a key regulator of binding equilibrium and kinetics of cell adhesion. Current techniques to examine the kinetics of cell adhesion are either performed in the absence of flow or are low throughput, limiting their application to pharmacological compound screening or the high throughput investigation of biological mechanisms. We developed a high throughput flow device that applies flow in a multi-well format and interfaced this system with electric cell-substrate impedance sensing (ECIS) system to allow label free detection of cell adhesion. We demonstrate that this combined system is capable of making real time measurements of cancer cell adhesion to extracellular matrix and immobilized platelets. In addition, we examined the dependence of the kinetics of binding of cancer cells on the level of shear stress and in the presence of small molecule inhibitors to adhesion-related pathways. This versatile system is broadly adaptable to the high throughput study of cell adhesion kinetics for many applications including drug screening and the investigation of the mechanisms of cancer metastasis.

Cancer metastasis is a cascade of multiple events leading to the formation of secondary tumors and is the underlying cause for the failure of therapy for most types of cancer. Many of the steps of metastasis are greatly influenced by the physical forces on the circulating tumor cells as they intravasate, disseminate to a new location, and then extravasate to form a secondary tumor^1^. The adhesion of cancer cells to the vasculature during extravasation is a key and limiting step for a cancer to colonize secondary sites within the body^2^. Several theories exist with respect to the mechanisms of this adhesion. Mechanical entrapment was originally proposed as a potential mechanism in comparison to the “seed and soil” theory of Paget^3^. However, the organ specificity and complex patterns of metastatic spread of different types of cancer support that specific mechanisms of adhesion are important beyond physical entrapment^4^. During extravasation, the initial attachment of circulating cancer cells to the target tissue is an essential step in the metastatic cascade that facilitates organ specific metastasis^5^. The specific mechanisms of adhesion are not completely understood but involve adhesion receptors of the selectin and integrin adhesion receptor families, as well as other cell surface receptors including CD44, CD164, and galectin-4^6^,^7^,^8^,^9^,^10^. The kinetics of receptor-ligand binding are key within the metastatic process as the adhesion event occurs under the kinetic limits of the circulatory flow.

In studying adhesion-mediated processes, there are many previous assays developed to examine cell adhesion^11^. The simplest adhesion assay measures the number of cells that adhere to an endothelial monolayer or extracellular matrix using fluorescently labeled cells. This type of assay has recently been expanded using nanopatterning to be able to examine combinations of extracellular matrix^12^. These assays can be performed in a high throughput format using multi-well plates or patterned chips. As these assays generally require washing away non-adherent cells they overlook differences in adhesion kinetics beyond the single time point examined. In contrast, parallel flow chambers have been used to examine cancer and other types of cell adhesion under flow^13^,^14^,^15^. Parallel flow chamber assays can be performed with controlled steady flow and have found utility for measuring real time adhesion kinetics and rolling, but in most cases require observation with a microscope dramatically reducing throughput.

Recent advances in biosensor systems have allowed the detection of cell adhesion without the need for fluorescent or other types of labeling. For kinetic measurements, a surface sensing technique is needed in order quantify adhered cells versus those remaining in suspension. Several modes of sensing have been explored including using electric cell-substrate impedance sensing (ECIS)^16^, quartz crystal microbalance (QCM)^17^,^18^, resonant waveguide grating^19^ (e.g. Epic System) as well as other evanescent field based biosensors^20^,^21^,^22^. Resonant waveguide grating biosensors use light propagation through a grate coupled with a waveguide that yields either reflected or transmitted light for detection. Cell impedance measurements are done by applying a small alternating current across an electrode array in the bottom of each well, creating a potential across the electrodes that is measured as impedance. As cells adhere to the electrode, they act as insulators and increase the impedance sensed. A major advantage of these systems is the ability to examine the kinetics of adhesion. Adhesion kinetics not only provide valuable information pertaining to how quickly cells adhere to a matrix but can also provide a measure of adhesion strength if used during detachment of cells. Label free cell adhesion assays have been used in high throughput studies under static conditions and some have been adapted to low throughput assays under flow but combined high throughput assays with flow are lacking. High throughput, flow-incorporating assays would be useful for the development of small molecule inhibitors cell adhesion during cancer metastasis and a broad range of other physiological process. End point readings alone are often not sufficient to observe changes in cell adhesion patterns in the presence of drug treatments and may be discordant with the *in vivo* situation where flow and kinetics of binding are key aspects of the metastatic cascade.

Our group has recently developed a high throughput system for applying flow to cells in a 96-well format^23^,^24^. The high throughput cone-and-plate (HT-CAP) device uses an array of cone-tipped shafts that rotate to create flow similar to a cone-and-plate viscometer. In this study, we have interfaced this flow system with a high throughput electric cell-substrate impedance sensing (ECIS) device to enable the performance of high throughput studies on the adhesion of cells under flow (Figs 1 and 2). While many methods have been developed to precisely study cell adhesion kinetics, none are able to measure adhesion kinetics in the presence of shear stress in a high throughput manner. We have used this system to study the time course of adhesion of cancer cells to extracellular matrix and platelets. In addition, we have used a detachment assay to measure the strength of adhesion and kinetics of cell release from the surface under varying levels of shear stress. Finally, we performed high throughput studies of the effect of small molecule inhibitors on the adhesion kinetics of breast cancer cells.

## Results

### Kinetics of adhesion of cancer cells to extracellular matrix (ECM) is dependent on the applied flow environment

We first examined the adhesion kinetics of cancer cells to different ECM molecules and the dependence of this binding on the level of applied shear stress. We measured adhesion of MDA-MB-231 and MCF-7 cancer cells to well plates coated with purified ECM components (collagen I, collagen II, collagen IV, vitronectin, fibronectin, laminin, and tenascin). We adhered the cancer cells under static conditions as well as 0.5, 1, 2, and 3 dynes/cm^2^ of shear stress (Fig. 3). There was a complex dependence of the adhesion on shear stress, cancer cell line and the ECM. Interestingly, there was a significant increase in adhesion at 1 dyne/cm^2^ of shear stress for all of the collagens, vitronectin and fibronectin for MDA-MB-231 cells. The MCF-7 cell line adhered most strongly to fibronectin under 1 or 2 dynes/cm^2^ of shear stress and both cell lines decreased their adhesion to laminin and vitronectin under shear stress. The adhesion curves were fit using a logistic function and the fit parameters compared by ECM type and applied shear stress (Supplemental Fig. 1).

### Pharmacological treatment with lysophosphatidic acid or paclitaxel alters cancer cell adhesion kinetics

Ultimately, a key goal of *in vitro* assays for cancer cell adhesion is to identify compounds that can treat or prevent the metastasis of cancer. Lysophosphatidic acid (LPA) is a modified phospholipid that is water soluble, serves as an active signaling molecule and has been linked to enhancing tumor growth, invasion and metastasis^25^,^26^. We treated MDA-MB-231 cancer cells with LPA and allowed them to bind one of four different ECMs (collagen I, collagen IV, laminin and vitronectin). We compared adhesion under static conditions to adhesion in the presence of 0.5 dynes/cm^2^ of shear stress, and saw a significant decrease in the adhesion of cancer cells after treatment with LPA in shear conditions with no or little effect in static assays (Fig. 4). For example, while there was no effect from LPA on adhesion to collagen IV under static conditions, there was a significant decrease in adhesion under shear stress compared to the no treatment control. Likewise, adhesion to vitronectin also had no significant change with LPA treatment under static conditions, while there was a significant decrease in adhesion at all three concentrations of LPA when shear stress was applied during adhesion. Adhesion to laminin was also effected by treatment with LPA in both static and shear adhesion conditions, but much fewer cells adhered overall under shear conditions.

We also examined the effects of paclitaxel, a common chemotherapeutic drug and inhibitor of microtubule depolymerization. We next treated MDA-MB-231 cancer cells with paclitaxel and measured adhesion to ECMs with and without flow (Fig. 5). For collagen I, we found that there was decreased adhesion with treatment with paclitaxel under static conditions. However, as the concentration was increased there was an increase in adhesion of the cells in static conditions. This trend was observed for all of the ECMs studied under the static adhesion assay. There was a significant decrease in adhesion under both static and shear conditions when adhered to collagen IV and there were significantly fewer cells that adhered under shear conditions in comparison to static cancer cell adhesion. Adhesion to both laminin and vitronectin were also decreased at later time points under shear stress. For vitronectin there was an increase in adhesion at early time points that correlated with increasing dose of paclitaxel in the static adhesion assay.

### Integrin dependence of cancer cell adhesion to platelets

Several prior studies have demonstrated that platelets can facilitate the dissemination of cancer and enhance extravasation^27^,^28^. We next examined the role of integrin inhibitors on MDA-MB-231 cancer cell adhesion to platelets. We used a small library of integrin inhibitors to study the mechanisms of cancer cell adhesion to immobilized platelets under static conditions and 0.5 dynes/cm^2^ of shear stress (Fig. 6). Under static conditions, there was a very marked effect of three inhibitors including pan-integrin inhibitory RGDS peptides, cilengitide (αvβ3/αvβ5 integrin inhibitors) and PF-562271 (FAK inhibitor). The cancer cells either adhered strongly in the presence of the integrin inhibitors or they did not. However, the same experiment conducted under shear conditions yielded a much more graded response. While RGDS peptides, cilengitide, and PF-562271 had the greatest effect on cancer cell adhesion there was also a significant decrease in adhesion with treatment of ATN-161 (α5β1 inhibitor) and TC-I 15 (α2β1 inhibitor) in comparison to the control group under shear stress. Almost all treatment conditions had significantly fewer cells adhered under shear conditions in comparison to static conditions. After adhesion, the cancer cells were detached with increasing applied shear stresses. Here, we saw RGDS peptides, cilengitide, ATN-161, and PF-562271 had significantly fewer cells remaining after 20 dynes/cm^2^ shear stress was applied, indicating that those integrin inhibitors decreased the strength of adhesion to the immobilized platelets.

### Kinetics of adhesion of cancer cells to membrane proteins from different organs

A hallmark of metastasis is tendency for particular types of cancer to colonize specific organs^29^,^30^. We explored the ability of breast cancer (MDA-MB-231) and glioblastoma (A172) cell lines in their ability to bind to membrane proteins isolated from specific organs. The rationale was that binding to these proteins was a key aspect of cancer invasion into specific organs and these cell lines may exhibit tropism for a particular organ’s cell surface proteins. The breast cancer and glioblastoma cell lines had differing adhesion profiles for the organ proteins that were altered by the presence of shear stress (Fig. 7). For both the breast and brain cancer cell lines under static and shear conditions, the cells adhered most to the membrane proteins isolated from skin. In the presence of shear stress, MDA-MB-231 cancer cells significantly adhered to pancreas lysate as well, in comparison the other membrane proteins. The A172 cell line showed minor differences in relative adhesion to the lysates under static conditions but had increased differential expression under shear stress.

## Discussion

The primary cause of mortality in cancer patients is the metastatic spread of disease to other organ systems of the body. Tumor cell adhesion during extravasation and invasion are key processes that control the ability of cancer to spread. While the rationale of targeting adhesion receptors or related pathways to prevent these processes is appealing, cancer therapeutics targeting adhesion receptors have not yet found success in clinical trials. The mechanisms of cancer cell adhesion during metastasis remain unclear and a major limitation in this area is a lack of high throughput assays for cell adhesion that can be carried out in the presence of the forces in circulatory flow. To address this need, our studies have validated the capability of a combined system that integrates a high throughput flow system and a label free detection system to perform accurate kinetic measurements of cancer cell adhesion under flow in the 96-well plate format.

While the primary goal of our studies was to validate a high throughput kinetic assay for cell adhesion under flow, our results also reveal the complex relationship of cancer cell adhesion to the applied flow environment. During cell adhesion from suspension or circulation, the applied forces can act upon the ligand-receptor pair to alter the kinetics of binding^31^. If the force acts in the direction of the conformation change to allow binding it enhances the rate the reaction. Conversely, if it opposes the conformational change for binding it will reduce the rate of reaction. Shear stress has been shown to act through both of these mechanisms in the cases of flow-mediated detachment^32^ or shear stress enhanced cell adhesion^33^. Our studies support that these effects depend upon the cancer cell type and extracellular matrix. A fascinating finding of our studies is the surprising complexity of these dependence of cell adhesion upon shear stress and the ECM. For instance, for cancer cell adhesion to collagen II the adhesion increased for MDA-MB-231 cells when comparing static conditions to 1 dynes/cm^2^ of flow. However, for the MCF-7 cell line the opposite occurred. Such complex relationships would be difficult to identify among the many possible combinations and supports the need for high throughput adhesion assays with flow. A similarly complex response has been shown previously in cancer cell adhesion assays that utilized a parallel plate flow chamber shear system to study adhesion of highly and less metastatic colon cancer cell adhesion to ECM^34^. As well, consistent with our findings, previous work has shown greater adhesion of cancer cells to collagen I as compared to collagen IV, fibronectin or laminin in the presence of shear stress^34^.

Our studies examining effect of pharmacologic inhibitors on cancer cell adhesion also revealed the complexity of issues in designing drugs to inhibit cancer cell adhesion to ECM or platelets. Prior studies support that lysophosphatidic acid is an enhancer of cancer metastasis but in our studies it decreased adhesion to laminin under static and collagen IV, laminin and vitronectin under shear stress. There is likely an optimal level of adhesion that permits cancer cells to leave the primary tumor, adhere under flow invade into the metastatic site^35^. Our studies support that LPA reduces cancer cell adhesion to ECM, which may enhance the ability of the cell to leave the primary tumor and invade the target tissue. The cytoskeletal inhibitor paclitaxel also modified adhesion in an intricate manner. With lower concentrations of paclitaxel under static conditions we observed maximal inhibition of adhesion with the lowest dose. Upon increasing the dose, the cancer cell adhesion increased and, in the case of vitronectin, became higher than the control group at early time points. Interestingly, a similar phenomenon has been reported with a different breast cancer cell line in which paclitaxel enhanced adhesion to gelatin under static conditions^35^. Our studies on integrin dependence of cancer cell binding to platelets supported that there were key differences in the integrins needed to adhere to cancer cell under flow versus static conditions. Under static conditions, adhesion was inhibited in almost a binary manner by αvβ5/αvβ3, pan-integrin or FAK inhibitors. However, a much more complex relationship was found for adhesion under flow with different inhibitors achieving only partial inhibition of adhesion. Under flow conditions, inhibitors of α5β1 and α2β1 prevented adhesion to some extent while these were not effective under static conditions. Platelets are thought to form aggregates with circulating cancer cells during metastasis through binding of integrins including αvβ3^36^. Additionally, integrin α5β1 has been linked to cancer cell interactions with thrombi^37^. Our findings are consistent with these prior studies and highlight the importance of including flow in adhesion assays to develop pharmacological inhibitors in order recapitulate the relevant biophysical environment under which adhesion occurs.

Finally, we examined the relative adhesion of cancer cells to the membrane proteins derived from several different types of organs. The primary purpose of the study was to examine whether there was a differential propensity for cancer cells to adhere to the membrane proteins from different organs during invasion. We found that there were many differences in the adhesion kinetics between the breast cancer line (MDA-MB-231) and the glioblastoma line (A172). For both static and flow-incorporating assays, the cell lines adhered most quickly to the skin membrane protein extracts. In fact, cutaneous metastasis is known to occur relatively frequently in breast cancer patients. In patients with metachronous contralateral breast cancer for less than 5 years the cumulative incidence of cutaneous metastasis is 13% and visceral metastasis 19%, both higher than the incidence of bone metastasis and brain metastasis^38^. In fact, the overall incidence of cutaneous metastasis in breast cancer patients is around 20%^39^. Thus, while this assay does not recapitulate the binding events related to extravasation, it provides insight into the relative adhesive properties of cancer cells to specific organ proteins.

Assays for cell adhesion have great utility in many fields including immunology, infectious disease, cardiovascular disease and cancer. The archetypal assay is one in which a cell circulating in suspension interacts with an adherent cell type that is most often of epithelial or endothelial origin. The hydrodynamic forces of the surrounding flow are critical factors that alter the chemical equilibrium and kinetics of receptor-ligand interactions that allow the circulating cell to adhere to the immobilized cell monolayer or underlying ECM. Moreover, measurement of adhesion kinetics can provide more in depth information on the mechanisms of the adhesion process, including an understanding of adhesion rates over different time scales and the determination of cooperative adhesion mechanisms. The system provides a high throughput platform for the measurement of cell adhesion kinetics under fluid flow and will afford an improved screening assay for examining the mechanisms of metastasis and developing pharmacological agents to prevent the spread of cancer.

## Methods

### Cell culture

Cancer cell lines were purchased from commercial sources (Cell Biolabs, Inc.). The cells were cultured in DMEM growth medium supplemented with 10% fetal bovine serum (FBS), L-glutamine and penicillin-streptomycin. All cells were cultured in a humidified incubator at 37 °C under a 5% CO_2_ atmosphere.

### High Throughput Cone and Plate Shear Device

**Figure 2:**
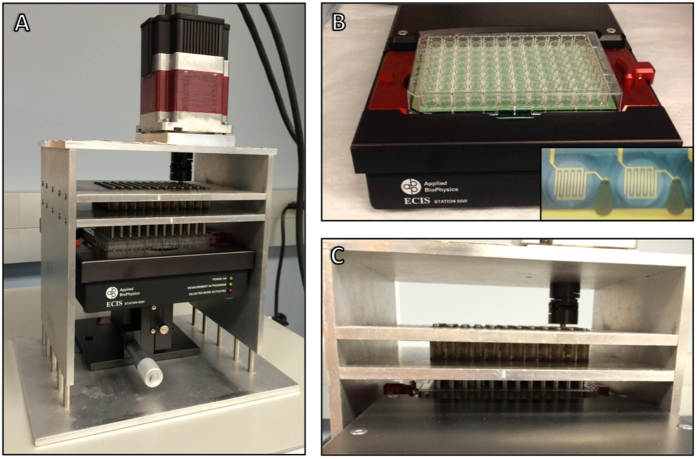
Images of the High Throughput Cone and Plate device interfaced with an Electric Cell-Substrate Impedance Sensing device. (**A**) The ECIS is placed on a stage lift to precisely position the well plate in relation to the low angle cone tips on the HT-CAP. Once positioned, the interfacing devices are placed in an incubator for temperature and CO_2_ control. (**B**) The well plates have a 40-electrode array on the bottom of each well. Once loaded onto the ECIS array station, electrical contact is made between the device and the well plate, enabling impedance measurements to be made. (**C**) Close-up image showing the low-angle cone tip shafts positioned in the wells of the 96-well electrode array plate that has been loaded into the ECIS device.

**Figure 3:**
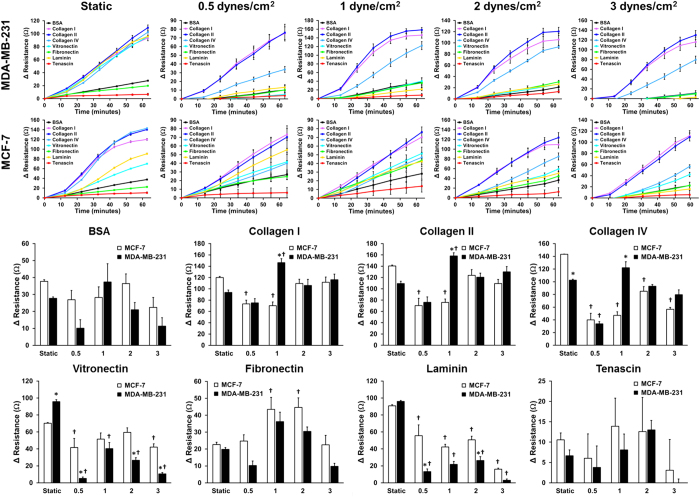
Kinetic adhesion of breast cancer cells to extracellular matrix under static or flow conditions. (A) Adhesion kinetic plots of the change in resistance over time for adhesion of metastatic MDA-MB-231 cells and less aggressive MCF-7 cells to extracellular matrix components under static conditions or 0.5, 1, 2 or 3 dynes/cm^2^ of applied shear stress. (B) Plots of adherent MDA-MB-231 and MCF-7 breast cancer cells after one hour of adhesion under static or shear conditions for each ECM component to which cancer cells were adhered (^*^p < 0.05 versus MCF-7 cells; ^†^p < 0.05 versus static conditions).

**Figure 4:**
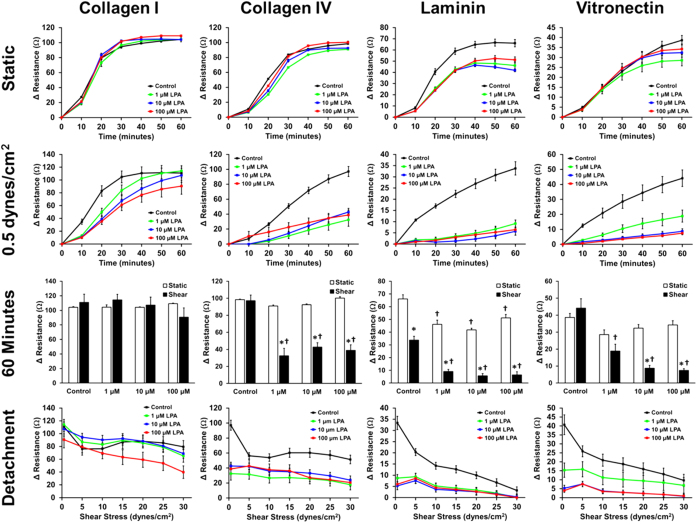
Dose response of the adhesion kinetics of metastatic cancer cells treated with LPA when adhered to ECM under static and flow conditions. MDA-MB-231 breast cancer cells pre-treated with either 0, 1, 10, or 100 μM of lysophosphatidic acid (LPA) were adhered to collagen I, collagen IV, laminin, or vitronectin under static conditions or 0.5 dynes/cm^2^ of shear stress for 60 minutes, and then detached at increasing shear stresses (^*^p < 0.05 versus static conditions; ^†^p < 0.05 versus control treatment).

**Figure 5:**
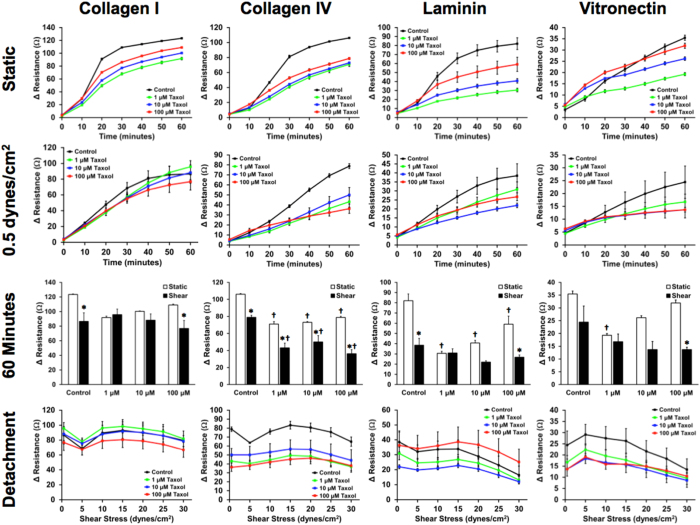
Dose response adhesion kinetics of metastatic breast cancer cells to paclitaxel when adhered to ECM under static and flow conditions. Metastatic MDA-MB-231 breast cancer cells were pre-treated with paclitaxel at concentrations of 0, 1, 10, and 100 μM, then adhered to ECM for 60 minutes under static and low shear stress (0.5 dynes/cm^2^) flow conditions. After the adhesion assay, a detachment experiment was performed on the adhered cells (^*^p < 0.05 versus static conditions; ^†^p < 0.05 versus control treatment).

**Figure 6:**
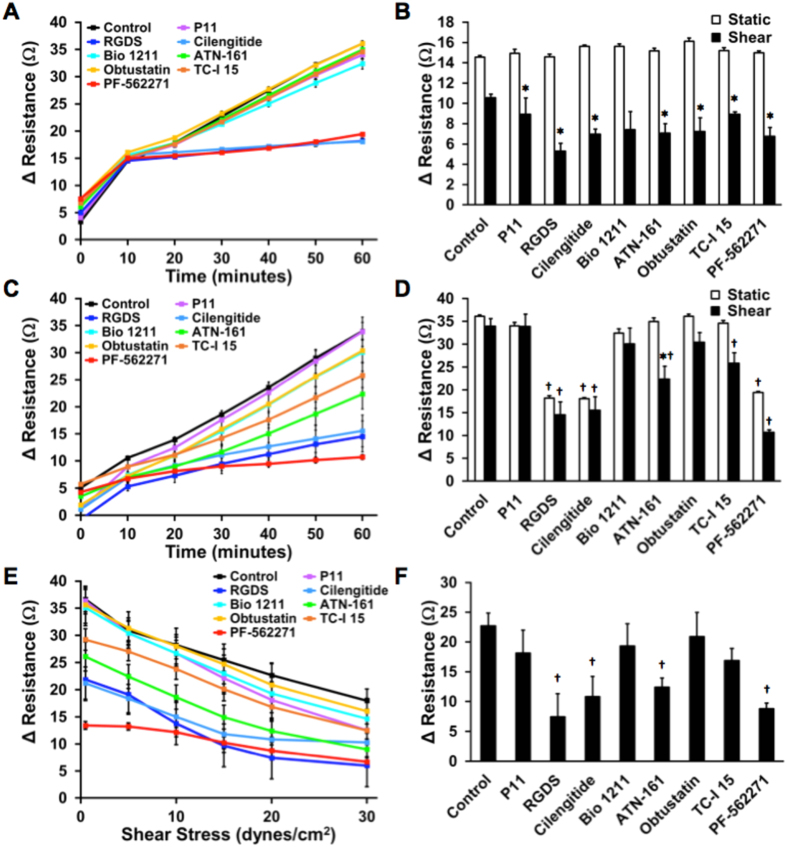
Screen of integrin inhibitors on kinetics of cancer cell adhesion to platelets under static and flow conditions. (**A**) Metastatic MDA-MB-231 cells were adhered to platelets in the presence of a small library of integrin inhibitors under static. (**B**) MDA-MB-231 cells were adhered to platelets under 0.5 dynes/cm^2^ of shear stress. (**C**) Change in resistance created by adherent breast cancer cells after 10 minutes of adhesion under static and flow conditions. (**D**) Final adhesion of breast cancer cells after 60 minutes of adhesion. (**E**) Detachment assay on adherent cancer cells at increasing shear stresses after treatment with a small library of integrin inhibitors. (**F**) Resistance of remaining cancer cells after 20 dynes/cm^2^ of shear stress is applied to detach cells (^*^p < 0.05 versus static conditions; ^†^p < 0.05 versus respective static or shear control treatment).

**Figure 7:**
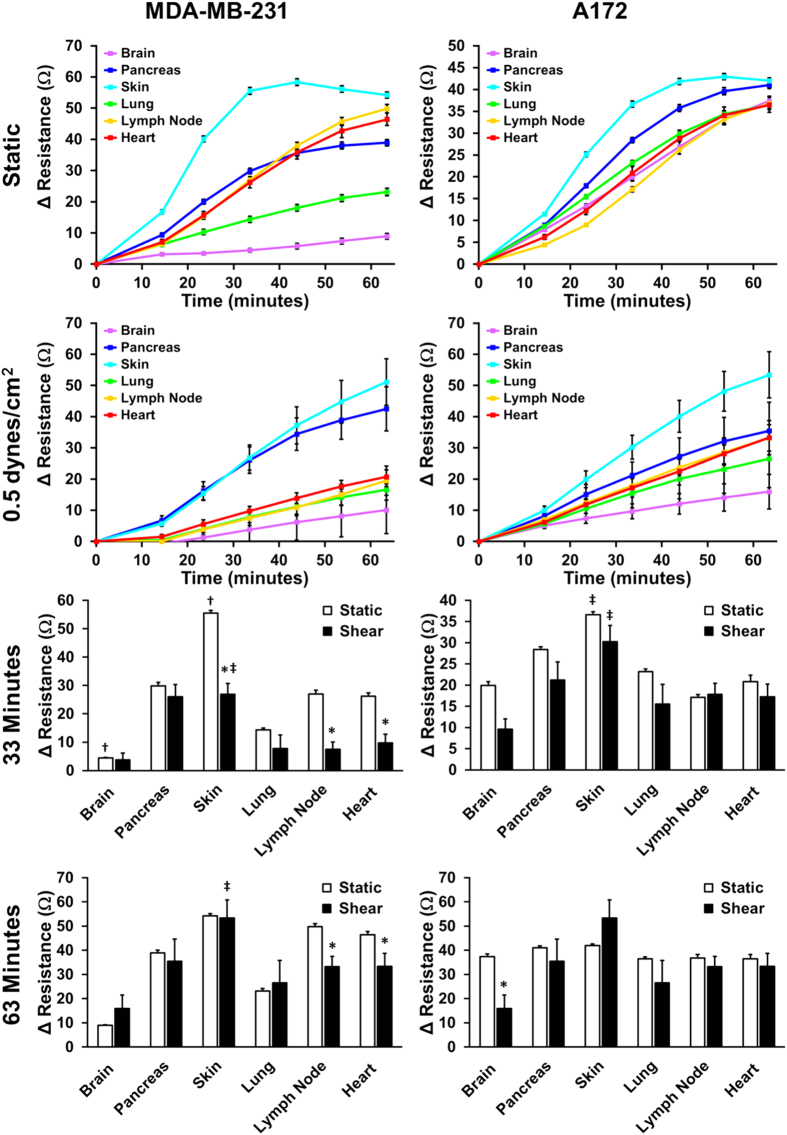
Adhesion kinetics of MDA-MB-231 breast cancer cells and A172 glioblastoma cells to human organ cell lysate. (A) MDA-MB-231 and A172 cancer cell adhesion kinetics under static conditions or 0.5 dynes/cm^2^ of shear stress. (B) Resistance of adherent cells after 30 minutes and 60 minutes of adhesion under static and flow conditions (^*^p < 0.05 versus static conditions; ^†^p < 0.05 versus all other lysate for respective static or shear conditions; ^‡^p < 0.05 versus all other lysate except pancreas for respective static or shear conditions).

A high throughput cone-and-plate device, designed in the lab was used to apply shear stress to a standard 96-well plate^24^. The device consists of a rotational motor that drives a shaft connected to a 96-gear box, thereby rotating all 96 shafts simultaneously (Fig. 2). Each shaft has a low angle cone-tip, creating a uniform shear stress on the surface of the well underneath the rotating shaft. The cone-tips were aligned with high precision and validated for biocompatibility and well-to-well variability in previous studies^24^.

### Electric Cell-Substrate Impedance Sensing (ECIS)

The high throughput ECIS Zθ system (Applied BioPhysics, Inc.) was used for measurements of adhesion. To measure resistance, well plates were used that contained gold electrode surfaces (Fig. 2B). These surfaces were coated with cysteine to stabilize the electrodes by treatment with 10 mM cysteine for 10 minutes. A 96 well plate with electrodes in the bottom of each well was placed in the well station, and resistance measurements were taken at a frequency of 40,000 Hz. During experiments, resistance, impedance, and capacitance readings were measured. As cells attached to the surface and spread the resistance readings increased.

### Cancer cell adhesion assay

Extracellular matrix, immobilized platelets or cell lysates were used to coat 96-well array plates. Extracellular matrix components used to coat the 96 well electrode array plates (Applied Biosystems) were used at the following concentrations: collagen I (110 μg/mL), collagen II (50 μg/mL), collagen IV (50 μg/mL), fibronectin (8 μg/mL), laminin (16.5 μg/mL), vitronectin (0.5 μg/mL) (Sigma), and tenascin C (8.25 μg/mL; R&D). For measurement of adhesion to platelets, platelet rich plasma was spun down in a 96-well plate to immobilize platelets to the bottom of the wells. In some studies, the membrane proteins from human lymph node, heart atrium (Abcam), liver, lung, brain, kidney, skin and pancreas (Novus Bio) were used to coat a 96-well electrode array plate by incubating overnight. Breast cancer cells were trypsinized and allowed to recover for 1 hour with mild agitation to prevent attachment. Breast cancer cells were added to the ECM-coated well plate at a concentration of 5 × 10^5^ cells/mL and the flow applied using the HT-CAP shear stress device for 60 minutes, and resistance measurements were taken at 10-minute intervals. The experiment was repeated under static conditions for comparison. In experiments where integrin inhibitors were used, the integrin inhibitors were added to the well plate directly before the addition of cancer cells, and then shear stress was applied. The integrin inhibitors were added at the following concentrations: P11 (25 nM), cilengitide (25 mM), ATN-161 (50 mM), TC-I 15 (2 mM), RGDS peptide (100 mM), Bio 1211 (4 nM), and obtustatin (2 nM). For all other drug treatments (paclitaxel and lysophosphatidic acid), cancer cells were treated with the drug for four hours prior to the experiment. In some cases, the remaining adherent cells were detached using increasing bouts of shear stress (5, 10, 15, 20, 25, and 30 dynes/cm^2^) in one-minute intervals each. After the each application of shear stress resistance measurements were recorded.

### Kinetic modeling of cell adhesion and detachment data

Kinetic data was fit to a logistic function using the following equation: 
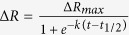
; where Δ*R* is the change in resistance, Δ*R*_max_ is the maximum change in resistance, k is a constant associated with the rate of adhesion, and *t*_*1/2*_ is the time to reach half of the maximum adhesion. A similar fit has been used to model cell adhesion under static conditions^40^,^41^.

### Statistical Analysis

All results are shown as mean ± standard error of the mean. Comparisons between only two groups were performed using a 2-tailed Student’s t-test. Differences were considered significant at p < 0.05. Multiple comparisons between groups were analyzed by 2-way ANOVA followed by a Tukey post-hoc test. A two-tailed probability value <0.05 was considered statistically significant.

## Additional Information

**How to cite this article**: Spencer, A. and Baker, A. B. High Throughput Label Free Measurement of Cancer Cell Adhesion Kinetics Under Hemodynamic Flow. *Sci. Rep.* **6**, 19854; doi: 10.1038/srep19854 (2016).

## Supplementary Material

Supplementary Information

## Figures and Tables

**Figure 1:**
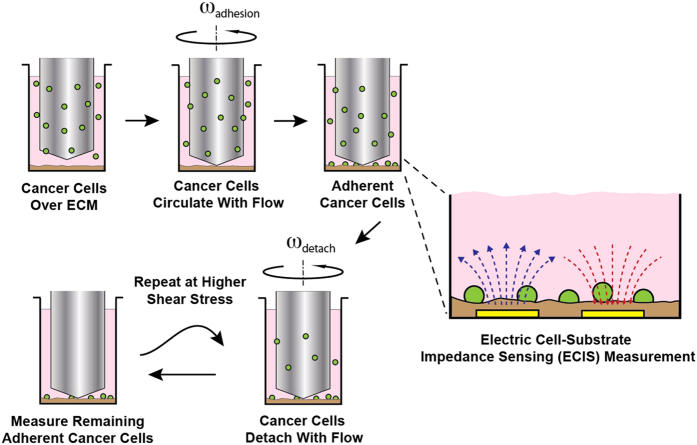
Label-free assay for cell adhesion-detachment under flow in a 96-well plate. Unlabeled cancer cells are added to the well plate, and a high-throughput cone-and-plate (HT-CAP) device is used to apply shear stress in the wells of a 96-well plate. Cell adhesion is measured with Electric Cell-Substrate Impedance Sensing (ECIS) measurements. Then higher shear stresses are applied to detach cancer cells and remaining cells are measured via ECIS.
