# Supplementary material for: High Throughput Label Free Measurement of Cancer Cell Adhesion Kinetics Under Hemodynamic Flow
Source: Sci Rep. 2016 Jan 27;6:19854. doi: 10.1038/srep19854 (PMC4728493; doi:10.1038/srep19854)
Supplement: Supplementary Information [file srep19854-s1.pdf]

Supplementary Information

High Throughput Label Free Measurement of Cancer Cell Adhesion Kinetics Under Hemodynamic Flow

Adrianne Spencer[1] and Aaron B. Baker[1,2,3]

[1]University of Texas at Austin, Department of Biomedical Engineering, University of Texas, Austin, TX

[2]Institute for Cellular and Molecular Biology, University of Texas, Austin, TX

[3]Institute for Computational Engineering and Sciences (ICES), University of Texas, Austin, TX

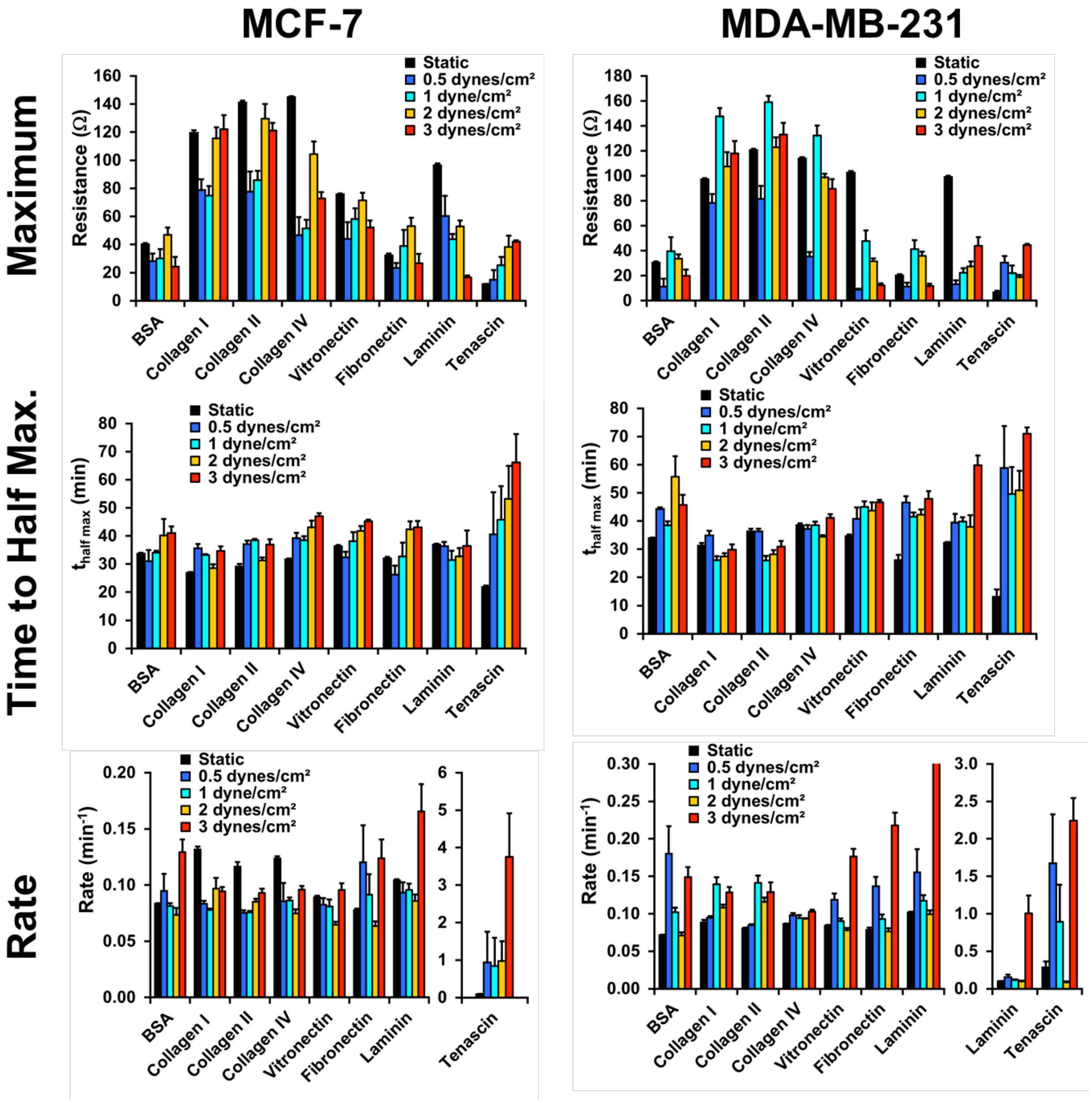

Supplemental Figure 1. Adhesion kinetics parameters for breast cancer cell adhesion to ECM under static and flow conditions. Less aggressive MCF-7 and metastatic MDA-MB-231 breast cancer cells were adhered to ECM under no flow or 0.5, 1, 2, or 3 dynes/cm^2 of shear stress for 60 minutes, then sigmoidal curves were fit to the adhesion data. The average fits for maximum adhesion as measured by resistance in the well, time to reach half of the maximum adhesion, and rate of adhesion were plotted for each shear condition for both MCF-7 and MDA-MB-231 cells.
